# Validation of causal inference data using DirectLiNGAM in an environmental small-scale model and calculation settings

**DOI:** 10.1016/j.mex.2023.102528

**Published:** 2023-12-20

**Authors:** Atsushi Kurotani, Hirokuni Miyamoto, Jun Kikuchi

**Affiliations:** a Research Center for Agricultural Information Technology, National Agriculture and Food Research Organization, Tsukuba, Ibaraki 305-0856, Japan; b Tokyo University of Agriculture and Technology, Koganei, Tokyo 184-0012, Japan; c Graduate School of Horticulture, Chiba University: Matsudo, Chiba 271-8501, Japan; d RIKEN Center for Integrated Medical Science, Yokohama, Kanagawa 230-0045, Japan; e RIKEN Center for Sustainable Resource Science, Yokohama, Kanagawa 230-0045, Japan

**Keywords:** LiNGAM, Field data, Correlation map, Feature importance, Causal effect, Treatment effect, DirectLiNGAM: A causal inference by direct estimation approach for learning the basic LiNGAM model with non-Gaussian data

## Abstract

The development of data science has been needed in environmental fields such as marine, weather, and soil data. In general, the datasets are large in some cases, but they are often small because they contain observation data that the analyses themselves are limited. In such a case, the data are statistically evaluated by increasing or decreasing the levels of factors using differential analysis, resulting in the essential factors are estimated. However, there is no consistent approach to the means of assessing strong associations as a group between factors. Causal inference method has the possibility to output effective results for small data, and the results are expected to provide important information for understanding the potential highly association between factors, not necessarily the inference with big data. Here, we describe essential checkpoints and settings for the calculation by a direct method for learning a linear non-Gaussian structural equation model (DirectLiNGAM) and validation methods for the calculation results by using DirectLiNGAM with small-scale model data as an additional discussion of DirectLiNGAM portion of the related research article. Thus, this study provides the statistical validation methods for the association networks, treatments, and interventions for structural inference as a group of essential factors.

• Causal inference with DirectLiNGAM
• Validation of correlation coefficient and feature importance
• Validation using causal effect object and propensity scores

## Graphical abstract

**Figure:**
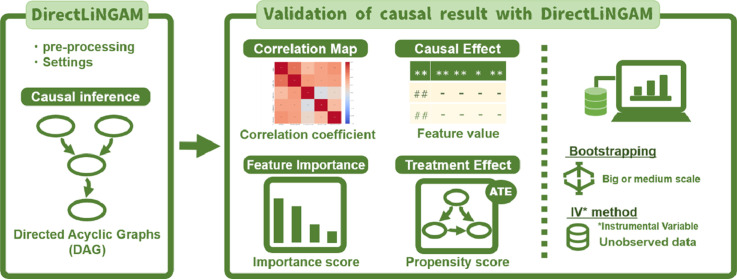

Specifications table

**Table:** 

Subject area:	Environmental Science
More specific subject area:	Marine Environmental Science
Name of your method:	DirectLiNGAM: A causal inference by direct estimation approach for learning the basic LiNGAM model with non-Gaussian data
Name and reference of original method:	DirectLiNGAM: A direct method for learning a linear non-Gaussian structural equation model, 2011, arXiv:1101.2489[2] Pairwise Likelihood Ratios for Estimation of Non-Gaussian Structural Equation Models, 2013, PMID: 31,695,580[3]
Resource availability:	Script: (DirectLiNGAM) https://github.com/cdt15/lingam https://lingam.readthedocs.io/en/stable/_modules/lingam/direct_lingam.html (Importance) https://github.com/VirajBagal/IEEE-Fraud/blob/master/feature-selection-using-null-importance.ipynb https://www.kaggle.com/code/ogrellier/feature-selection-with-null-importances (Causal Effect from LiNGAM tutorial) https://lingam.readthedocs.io/en/stable/reference/causal_effect.html Original data: Related research article link https://doi.org/10.1016/j.envres.2022.115130 — Supplementary data of related research article [1], https://ars.els-cdn.com/content/image/1-s2.0-S0013935122024574-mmc2.xlsx, sheet name:DS2ex for ELA

## Brief information

The Linear Non-Gaussian Acyclic Model (LiNGAM) algorithm, which is a causal direction estimation method using independent component analysis, was developed by the Shimizu et.al. in 2006 [4]. Afterwards, DirectLiNGAM, which is a direct, efficient, and stable estimation method for LiNGAM, was developed [2,5,6]. Pairwise-based DirectLiNGAM, which uses a simple linear approximation to reduce the computational cost of the likelihood ratio, was later developed [3]. DirectLiNGAM has been applied to various data, such as medical and health data [7,8], metagenome and metabolome data [9,10], and financial data [11]. The background information is detailed in the ‘Additional Information’ section.

## Method details

In the related research article [1], causal inference by a direct estimation approach based on non-Gaussian data using the DirectLiNGAM framework was performed as one analytical method for understanding the relationships among symbiotic bacterial groups. In this paper, we describe the detailed use of DirectLiNGAM in the above paper and validate the results considering future generic applications.

### Preparation and calculation setting of DirectLiNGAM

#### Dataset and data preprocessing

The entire dataset, including 18 records and 51 items, comprises the results of metagenomic analysis of symbiotic bacterial communities collected from marine soils on the coasts under both seagrass-abundant areas and nonabundant areas. During data preprocessing for creating the model dataset, all data items were first narrowed down to a minimum of 5 items, including the outcome variable with a very likely causal relationship to seagrass, by considering the affinity between the factors based on the results of association analysis (AA), linear discrimination analysis (LDA), [1] energy landscape analysis (ELA) [1,12], and structural equation modelling (SEM) [1,13]. After item refinement, the data were used in DirectLiNGAM for causal inference. The data were standardized through preprocessing.

#### Program language

DirectLiNGAM is written in the Python language.

#### Operating system and Python versions

We confirmed the operating systems, including Windows10, 11, CentOs7.6, MacOS10–13, and Python version from 3.6 to 3.11, the detailed versions of which are 3.6.4, 3.7.3, 3.8.5, 3.8.13, 3.9.1, 3.10.9, and 3.11.4, respectively. When the library versions and calculation settings in the DirectLiNGAM program were the same, the use of different OSs and Python versions did not affect the results.

#### Required libraries, library versions, and differences in library versions

**Fig. 1:**
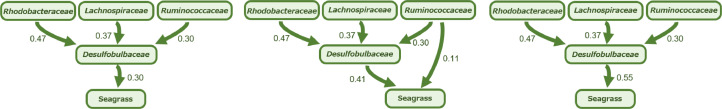
Network diagram of seagrass and soil bacteria from the sea using DirectLiNGAM causal inference with several versions of scikit-learn. In this calculation, the data standardization process is performed, the pairwise method is used as the measurement method, and the cut-off value of the adjacency matrix is not set or set to 0.1. When the version of scikit-learn library is case (A) or (C), the network structure is the same. On the other hand, when the version is case (B), the network structure is slightly different. However, if the cut-off value is slightly higher (e.g., 0.2 or higher), all of cases (A)-(C) become the same structure (data not shown).

To run the DirectLiNGAM program, Python libraries, including the LiNGAM library for the DirectLiNGAM operation, the graphviz library for network data visualization, and the NumPy and pandas library for data processing and computation, must be imported. In addition, scikit-learn, which is a data scientific library, is necessary for data standardization, such as clustering, scaling and regression, and running DirectLiNGAM. These libraries currently have various versions. In this paper, we used v1.8.1 for LiNGAM, v0.20.1 for graphviz, v1.22.4 for NumPy, and v1.4.3 for pandas. Notably, the use of different versions of the LiNGAM, graphviz, NumPy, and pandas library in the processing of DirectLiNGAM do not affect the results. However, the different scikit-learn library versions, which include v0.22.0 (the oldest currently available version)-v1.0.1, v1.0.2-v1.1.3, and v1.2.0-v1.3.0 (the newest currently available version), exhibit somewhat different network structures and distance values on the same data and program (Fig. 1). We used scikit-learn v1.22.4 in the related research article [1] and in the following sections of this paper.

#### Causal order determination method in calculation setting

In the DirectLiNGAM program, the ‘pwling’ and ‘kernel’ measurement methods are available. ‘Pwling’ is a pairwise method that is the default in the program and uses simple first-order approximations of the likelihood ratio to reduce computational burden [3,7], while ‘kernel’ is a direct estimation method that uses regression analysis from multivariate data, is more sensitive to noise and may be less accurate [2,3]. The results of DirectLiNGAM using kernel measurement is shown in Fig. S1.

### Validation of data results

#### Correlation coefficient

In general, the trends in the affinity between the correlation coefficient and causal estimation are not identical, but they are relatively similar. Therefore, the correlation coefficients found by the best-subset selection procedure calculated with Pearson's method, which is a parametric test, and Spearman's method, which is a nonparametric test with rank-correlations, can be found as one of a causal inference validation method.

**Fig. 2:**
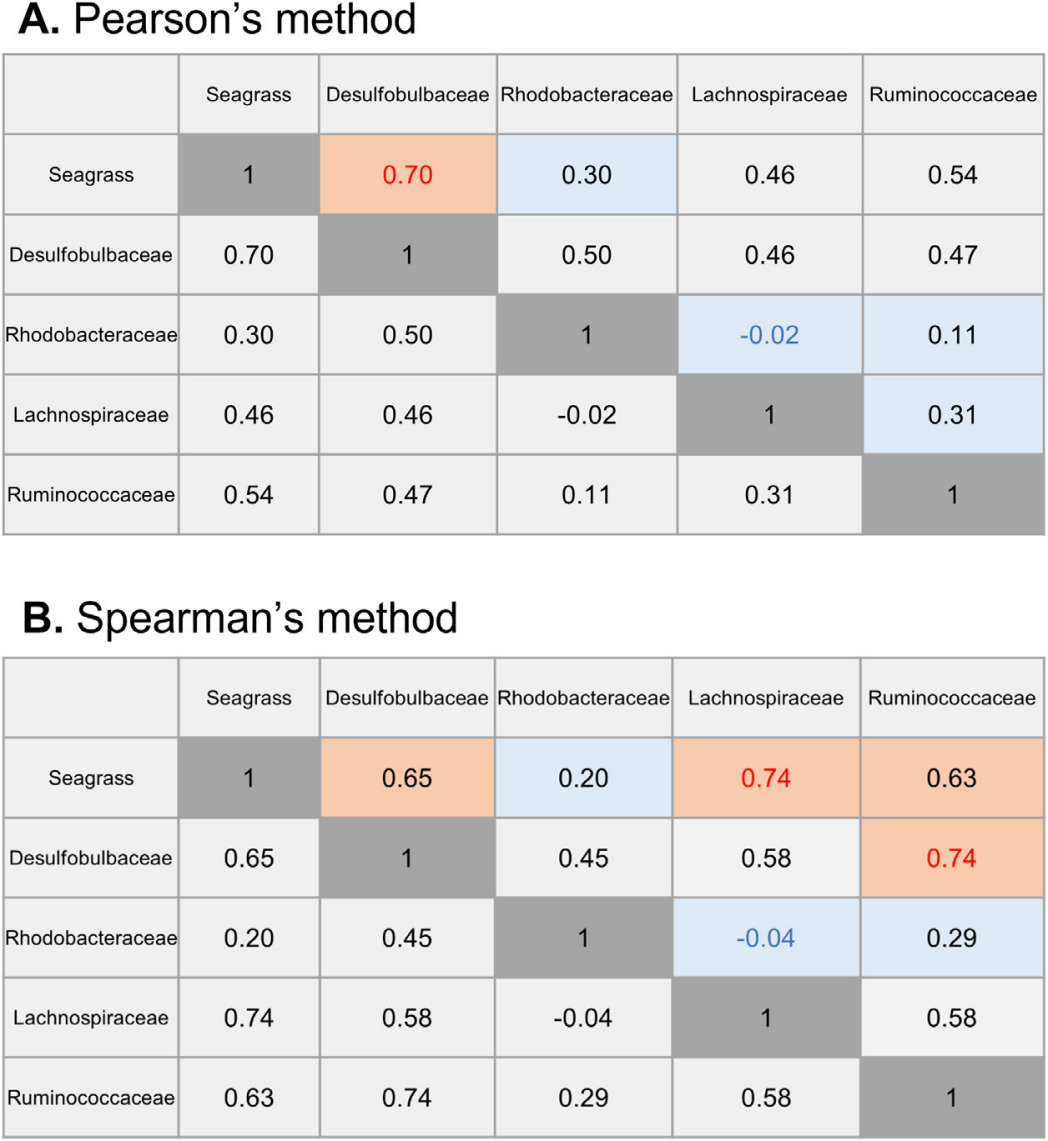
Heatmap of Correlation coefficients with Pearson's and Spearman's method by best-subset selection procedure. The upper figure is the result of Pearson's method, while lower figure is the result of Spearman's method. The colors in the heatmap are classified as red letter≧0.7, orange≧0.6, 0.6>lightgray>0.3, 0.3≧lightblue, and 0>blue letter. These are described using half of the columns.

As a result of using the dataset, the correlation coefficient with Pearson's method was high for *Desulfobulbaceae* against seagrass, the correlation coefficients for *Desulfobulbaceae* against other factors were relatively mediocre, and the correlation coefficients for *Rhodobacteraceae, Lachnospiraceae* and *Ruminococcaceae* were low (Fig. 2A). Overall, the affinity trends with correlation coefficients were similar to the relationship in the DirectLiNGAM network. Further, although using the Spearman method resulted in somewhat different results than using the Pearson method, although the difference was minor (Fig. 2B). The source code of the applicable section for obtaining the correlation coefficient is as follows. df = pd. DataFrame (designated data) df.cor(method='spearman' | 'pearson')

#### Feature importance score

Moreover, as one of the validations of causal inference, the importance scores (e.g., null importance) between each item for the target variable can be identified. The importance scores can be obtained if the ‘feature_importances_’ function is supported by the learning model (e.g., random forest (RF), XGBoost, LightBGM, etc.) in the Python program. In the null importance method, the importance scores between actual importances with actual training data and null importances with training data generated by shuffling objective variables are calculated. The source codes of the importance calculating program can be accessed at the URL provided in the Specifications table.

**Fig. 3:**
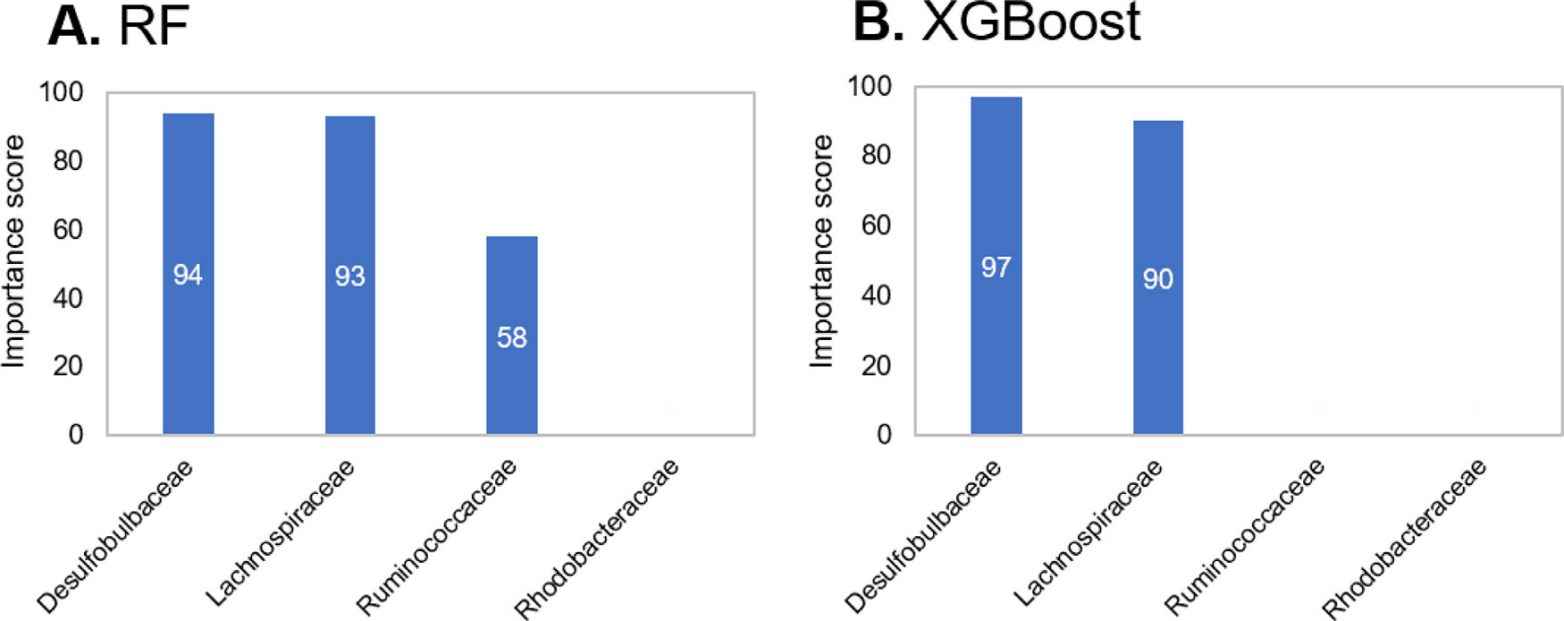
Calculation of importance score with null importance method.

The importance scores of *Desulfobulbaceae* and *Lachnospiraceae* for seagrass, calculated as objective variables using the dataset, were high, while the other importance scores were lower or zero (Fig. 3). Therefore, the affinity results of the importance scores are similar to the causality results of DirectLiNGAM.

#### Validation of intervention effect with LiNGAM ‘CausalEffect’ object

(Maximum feature value)

The estimated values of the intervention effect using ‘estimate_effects_on_prediction’, which is one of the functions of the causal effect in the DirectLiNGAM model in the LiNGAM Python library, can be used as a causal inference validation method. In this calculation, the data should not be standardized because the prediction values are specified after intervention.

To obtain the results, we used several prediction models, including linear regression, RF regression and XGBoost regression, with this function (Table 1). Notably, the linear regression model is used as the prediction model in LiNGAM's causal effect tutorial. Here, the item with the largest absolute value in the estimated values, the max feature value, is defined as the feature with the largest intervention effect in this algorithm. Incidentally, various other learners (e.g., LightBGM, logistic regression, LassoCV, ridge, MLP regression, KNeighbors regression) can be applied to the prediction model of this function. The source code for obtaining the feature value estimation is as follows and can be accessed at the URL in the causal effect section of the Specifications table.

**Table 1:** Estimation of feature values for seagrass as target data. Effect (plus) is calculated by E[Y|do(Xi=mean)]-E[Y|do(Xi=mean+std)], and Effect (minus) is calculated by E[Y|do(Xi=mean)]–E[Y|do(Xi=mean-std)]. Y is seagrass data as target data, Xi is the data for each explanatory variables, and do means the processing of intervention operations. The brackets under ‘Max feature value’ indicate the optimal intervention value.

	Linear Regression		RF Regression		XGBoost Regression	
	effect (plus)	effect (minus)	effect (plus)	effect (minus)	effect (plus)	effect (minus)
Desulfobulbaceae	**0.247**	**−0.247**	0.320	−0.180	**0.574**	−0.003
Rhodobacteraceae	0.132	−0.132	0.270	−0.290	0.556	−0.452
Lachnospiraceae	0.168	−0.168	0.290	**−0.430**	0.547	−0.511
Ruminococcaceae	0.202	−0.202	0.230	−0.280	0.546	−0.381
Max feature value	Desulfobulbaceae (0.131)		Lachnospiraceae (0.029)		Desulfobulbaceae (0.131)	

lce = lingam.CausalEffect(lingam. DirectLiNGAM())

lce.estimate_effects_on_prediction(X*, target_index*, prediction_model*)

*X: training data

*target_index: target variable (seagrass)

*prediction_model: Linear regression, RF regression, XGBoost regression

According to this intervention effect calculation, the maximum feature values for the target were achieved by *Desulfobulbaceae* in the linear regression and XGBoost regression methods and *Lachnospiraceae* in the RF regression method. Therefore, the effect of these bacteria on seagrass could also be considered.

(Optimal intervention value in maximum feature value)

According to the above results, the maximum feature value for the intervention effect is obtained by *Desulfobulbaceae* or *Lachnospiraceae*. In addition, the optimal intervention value in the maximum feature value using the ‘estimate_optimal_intervention’ function in the causal effect of the DirectLiNGAM model can be calculated [14]. In this process, linear regression is used as the prediction model because the function is supported by only a linear model. Ridge and LassoCV, which are also linear models, can also be used for this process. The optimal intervention value in *Desulfobulbaceae* was calculated as 0.131 when the maximum feature value was *Desulfobulbaceae* and the desired output was 1. Namely, for data interpretation, the observed value of *Desulfobulbaceae* must be 0.131 or higher for the occurrence of seagrass. Similarly, the observed value of *Lachnospiraceae* must be 0.029 or higher for the occurrence of seagrass. The source code for obtaining the optimal intervention value is as follows.

lce = lingam.CausalEffect(lingam. DirectLiNGAM())

lce.estimate_optimal_intervention(X, target_index, prediction_model*, intervention_index*, desired_output*)

*prediction_model: Linear regression

*intervention_index: Index of variable to apply intervention

*desired_output: desired expected postintervention output of prediction

#### Validation using propensity scores

The causal inference can be validated using propensity scores, score matching and stratified analysis. These are more effective than simple numerical comparisons for estimating treatment effects without bias. In addition, as other validation methods using propensity scores, the inverse probability weighting (IPW) and double robustness (DR) methods can also be used to verify the average treatment effect (ATE). These methods require a certain propensity score accuracy because propensity score errors often significantly influence the results. Therefore, the distribution of propensity scores should be unbiased. ATE is a widely used indicator; the other indicators ATT, which is the average treatment effect on treated samples, and ATUm which is the average treatment effect on untreated samples.

Using the dataset, we verified the ATE with the IPW and DR method. To classify the data, considering the network diagram in Fig. 1, the occurrence of seagrass was considered to be the outcome, the item *Desulfobulbaceae* was the treatment variable, and the items *Lachnospiraceae, Ruminococcaceae* and *Rhodobacteraceae* were explanatory variables employed as covariates for the occurrence of seagrass. Here, the values of *Desulfobulbaceae*, which are treatment variables and are continuous values, were represented as 0 and 1, which indicate scores below-median and above-median, respectively. Logistic regression was used to calculate the propensity score, and linear regression was used to calculate the counterfactual model in the DR method. Learners are widely used for this process [15], [16], [17]. The AUC-ROC for propensity score estimation using the logistic regression model was 0.667. The AUC-ROC values for the ATEs calculated with the IPW and DR methods were 0.776 and 0.708, respectively (Table 2). This result indicates that the presence of *Desulfobulbaceae* positively impacts the occurrence of seagrass.

**Table 2:** ATE value for each estimation method using propensity score.

Method	ATE
IPW	0.776
DR	0.708

#### Other validations

Bootstrapping [18], which uses random sampling iterations to raise accuracy and is a function of the DirectLiNGAM platform, is an effective method for validating the causal structure. An example code for the applicable portion of the bootstrapping program is as follows. model = lingam. DirectLiNGAM() model.bootstrap(df, n_sampling=1000 | 2000 | 3000 | 4000 | 5000)

Notably, the credibility of the results obtained by bootstrapping is uncertain when the data size is small, especially when finding a confidence interval.

In addition, if unobserved data, such as confounding factors, are included in the causal inference data, the effect can be validated by the instrumental variable (IV) method with certain conditions.

## Conclusion

Considering causal inference methods, it is important to check their calculation methods and evaluate their results [19,20]. In this paper, we confirmed the basic usage of DirectLiNGAM and validated the result of causal inference with DirectLiNGAM. In addition, as validations for confirming credibility, we validated the correlation coefficients between factors, feature importance for the target variable, intervention effect and optimal value for the effect, and investigation with propensity score as effective methods even for small-scale data. Although various methods of path analysis have been reported [13,20,21], DirectLiNGAM provides a method with new point of view.

In recent years, DirectLiNGAM derivative tools have been developed, such as qLiNGAM [22], which uses a quantum kernel for the independence measure, and ParaLiNGAM [23], which accelerates DirectLiNGAM through parallel processing. The DirectLiNGAM-based causal inference tool has great potential for further evolution and potential future applications [24,25].

Furthermore, since it is not intended to calculate reliable causal relationships and numerical values, causal inference and verification methods for the result of DirectLiNGAM such as this paper can be applied to estimate a potential key group within environmental data [26]. Based on these perspectives, we hope that DirectLiNGAM will lead to solutions to various environmental problems.

## Ethics statements

This work did not involve human subjects, animal experiments data, and data collected from social media platforms.

## CRediT authorship contribution statement

**Atsushi Kurotani:** Methodology, Validation, Writing – original draft. **Hirokuni Miyamoto:** Validation, Writing – review & editing. **Jun Kikuchi:** Software, Writing – review & editing, Project administration, Supervision.

## Declaration of Competing Interest

The authors declare that they have no known competing financial interests or personal relationships that could have appeared to influence the work reported in this paper.

## Data Availability

The data that has been used is confidential.

## Appendix Supplementary materials

Image, application 1

Image, application 2
