# Supplementary material for: Validation of causal inference data using DirectLiNGAM in an environmental small-scale model and calculation settings
Source: MethodsX. 2023 Dec 20;12:102528. doi: 10.1016/j.mex.2023.102528 (PMC10809110; doi:10.1016/j.mex.2023.102528)
Supplement: Supplementary file 1 [file mmc1.docx]

**Supplementary material**

**
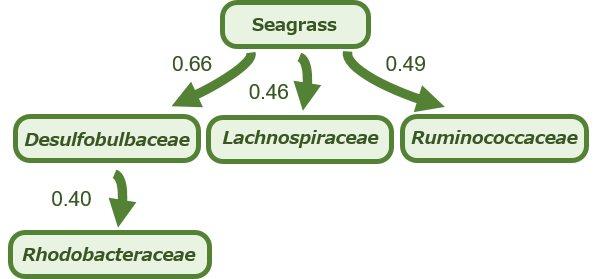
**

Fig. S1. Network diagram of seagrass and soil bacteria from the sea using DirectLiNGAM causal inference computed by the kernel measurement method. The seagrass starting position in the network structure differs significantly from that of the method using kernel measurement. The source code for the measurement method in the program is as follows:

lingam. DirectLiNGAM(measure = 'pwling' | 'kernel')
