# Supplementary material for: Validation of causal inference data using DirectLiNGAM in an environmental small-scale model and calculation settings
Source: MethodsX. 2023 Dec 20;12:102528. doi: 10.1016/j.mex.2023.102528 (PMC10809110; doi:10.1016/j.mex.2023.102528)
Supplement: Supplementary file 2 [file mmc2.docx]

**Additional information**

**Article title**

**Validation of causal inference data using DirectLiNGAM in an environmental small-scale model and calculation settings**

**Authors**

Atsushi Kurotani^a,b,*^, Kenta Suzuki^c^, Hirokuni Miyamoto^e,f,g,h,*^, Jun Kikuchi^d^

**Affiliations**

*^a^ Research Center for Agricultural Information Technology, National Agriculture and Food Research Organization, Tsukuba, Ibaraki, 305-0856, Japan*

*^b^ Tokyo University of Agriculture and Technology, Koganei, Tokyo 184-0012, Japan*

*^c^ RIKEN, BioResource Research Center, Tsukuba, Ibaraki, 305-0074, Japan*

*^d^ RIKEN Center for Sustainable Resource Science, Yokohama, Kanagawa, 230-0045, Japan*

*^e^ Graduate School of Horticulture, Chiba University: Matsudo, Chiba, 271-8501, Japan*

*^f^ RIKEN Center for Integrated Medical Science, Yokohama, Kanagawa, 230-0045, Japan*

*^g^ Japan Eco-science (Nikkan Kagaku) Co. Ltd.: Chiba, Chiba, 263-8522, Japan*

*^h^ Sermas Co., Ltd.: Ichikawa, Chiba, 272-0033, Japan*

**Corresponding author’s email address and Twitter handle**

[h-miyamoto@faculty.chiba-u.jp](mailto:h-miyamoto@faculty.chiba-u.jp), kurotania001@naro.affrc.go.jp

**Detailed background information**

Structural equation modelling (SEM) has long been used as an effective statistical method for calculating correlations and causal relationships among variables using datasets such as observations [1]. However, SEM is based on the Gaussian distribution dataset and statistical testing of hypothetical causal models based on comparing sample covariance and intrinsic covariance matrices (generally using the covariance structure), so the quality of the causal structure is not guaranteed. In addition, many datasets, such as those composed of observational data, have non-Gaussian data structures. Therefore, in 2006, the Shimizu group created the linear non-Gaussian acyclic model (LiNGAM) algorithm, which is a causal direction estimation method using independent component analysis (ICA) [2]. This algorithm is an extension of SEM that requires linear acyclicity in the data generation process and a non-Gaussian distribution with nonzero variance for the exogenous variables. Specifically, the algorithm has a non-Gaussian distribution as a characteristic property so that the variables are uncorrelated and each component can be distinguished from the others. As a result, the algorithm is represented by a directed acyclic graph (DAG) that allows semiparametric causal estimation. As a structure-finding algorithm of LiNGAM, DirectLiNGAM was developed [3,4]. This is a method for determining the causal order and coefficient matrix to minimize the correlations between error terms by conducting repeated single regression analysis between two variables and evaluating the independence of the error terms and avoids local optimization. Pairwise-based DirectLiNGAM, which uses a simple linear approximation to reduce the computational cost of the likelihood ratio, was later developed [5]. DirectLiNGAM has been applied in various domains, such as causal analysis of disease progression factors and health indicators with medical data and health checkup data [6,7], characteristic factor analysis in biochemistry fields, such as metagenomes and metabolomes [8,9], and causal analysis with financial data [10].

**References**

[1] K.A. Bollen, Structural equations with latent variables, Wiley, New York, 1989.

[2] P.O.H. Shohei Shimizu, Aapo Hyv¨arinen, Antti Kerminen, A Linear Non-Gaussian Acyclic Model for Causal Discovery, Journal of Machine Learning Research. 7 (2006) 2003-2030.

[3] S. Shimizu, A. Hyvärinen, Y. Kawahara, A direct method for estimating a causal ordering in a linear non-Gaussian acyclic model, 2009, pp. 506-513.

[4] T.I. Shohei Shimizu, Yasuhiro Sogawa, Aapo Hyv¨arinen, Yoshinobu Kawahara, TakashiWashio, Patrik O. Hoyer, Kenneth Bollen, DirectLiNGAM: DirectLiNGAM A Direct Method for Learning a Linear Non-Gaussian Structural Equation Model, Journal of Machine Learning Research. 12 (2011) 1225-1248.

[5] A. Hyvarinen, S.M. Smith, Pairwise Likelihood Ratios for Estimation of Non-Gaussian Structural Equation Models, J Mach Learn Res. 14 (2013) 111-152.

[6] T. Uchida, K. Fujiwara, K. Nishioji, M. Kobayashi, M. Kano, Y. Seko, K. Yamaguchi, Y. Itoh, H. Kadotani, Medical checkup data analysis method based on LiNGAM and its application to nonalcoholic fatty liver disease, Artif Intell Med. 128 (2022) 102310, doi:10.1016/j.artmed.2022.102310.

[7] J. Kotoku, A. Oyama, K. Kitazumi, H. Toki, A. Haga, R. Yamamoto, M. Shinzawa, M. Yamakawa, S. Fukui, K. Yamamoto, T. Moriyama, Causal relations of health indices inferred statistically using the DirectLiNGAM algorithm from big data of Osaka prefecture health checkups, PLoS One. 15 (2020) e0243229, doi:10.1371/journal.pone.0243229.

[8] S. Okada, Y. Inabu, H. Miyamoto, K. Suzuki, T. Kato, A. Kurotani, Y. Taguchi, R. Fujino, Y. Shiotsuka, T. Etoh, N. Tsuji, M. Matsuura, A. Tsuboi, A. Saito, H. Masuya, J. Kikuchi, Y. Nagasawa, A. Hirose, T. Hayashi, H. Ohno, H. Takahashi, Estimation of silent phenotypes of calf antibiotic dysbiosis, Sci Rep. 13 (2023) 6359, doi:10.1038/s41598-023-33444-0.

[9] H. Miyamoto, K. Shigeta, W. Suda, Y. Ichihashi, N. Nihei, M. Matsuura, A. Tsuboi, N. Tominaga, M. Aono, M. Sato, S. Taguchi, T. Nakaguma, N. Tsuji, C. Ishii, T. Matsushita, C. Shindo, T. Ito, T. Kato, A. Kurotani, H. Shima, S. Moriya, S. Wada, S. Horiuchi, T. Satoh, K. Mori, T. Nishiuchi, H. Miyamoto, H. Kodama, M. Hattori, H. Ohno, J. Kikuchi, M.Y. Hirai, An agroecological structure model of compost-soil-plant interactions for sustainable organic farming, ISME Commun. 3 (2023) 28, doi:10.1038/s43705-023-00233-9.

[10] M.R.F. Ivy Luo, Sandro Claudio Lera, Causal Analysis of Stakeholder Interdependencies in 10-K Reports, SSRN: <http://dx.doi.org/10.2139/ssrn.4483287> (2023) 19 pages.
